# SGLT2i treatment during AKI and its association with major adverse kidney events

**DOI:** 10.3389/fphar.2024.1356991

**Published:** 2024-06-12

**Authors:** Luz Alcantar-Vallin, Jose J. Zaragoza, Bladimir Díaz-Villavicencio, Karla Hernandez-Morales, Jahir R. Camacho-Guerrero, Miguel A. Perez-Venegas, Edgar J. Carmona-Morales, Alexa N. Oseguera-Gonzalez, Cesar Murguia-Soto, Gael Chávez-Alonso, José M. Arredondo-Dubois, Carlos E. Orozco-Chan, Juan A. Gómez-Fregoso, Francisco G. Rodríguez-García, Guillermo Navarro-Blackaller, Ramón Medina-González, Alejandro Martínez Gallardo-González, Gabriela J. Abundis-Mora, Olynka Vega-Vega, Guillermo García-García, Jonathan S. Chávez-Iñiguez

**Affiliations:** ^1^ Nephrology Service, Hospital Civil de Guadalajara Fray Antonio Alcalde, Guadalajara, Jalisco, Mexico; ^2^ University of Guadalajara Health Sciences Center, Guadalajara, Jalisco, Mexico; ^3^ Intensive Care Unit, Hospital H+ Queretaro, Queretaro, Mexico; ^4^ Departamento Nefrología y Metabolismo Mineral, Instituto Nacional de Ciencia Médicas y Nutrición Salvador Zubirán, Mexico City, Mexico

**Keywords:** acute kidney injury, sodium–glucose transporter type 2 inhibitor, major adverse kidney events, death, kidney replacement therapy

## Abstract

**Background:**

The association between the administration of sodium–glucose cotransporter 2 inhibitors (SGLT2is) during acute kidney injury (AKI) and the incidence of major adverse kidney events (MAKEs) is not known.

**Methods:**

This retrospective cohort study included patients with AKI and compared the outcomes for those who were treated with SGLT2is during hospitalization and those without SGLT2i treatment. The associations of SGLT2i use with MAKEs at 10 and 30–90 days, each individual MAKE component, and the pre-specified patient subgroups were analyzed.

**Results:**

From 2021 to 2023, 374 patients were included in the study—316 without SGLT2i use and 58 with SGLT2i use. Patients who were treated with SGLT2is were older; had a greater prevalence of diabetes, hypertension, chronic heart failure, and chronic kidney disease; required hemodialysis less often; and presented stage 3 AKI less frequently than those who were not treated with SGLT2is. Logistic regression analysis with nearest-neighbor matching revealed that SGLT2i use was not associated with the risk of MAKE10 (OR 1.08 [0.45–2.56]) or with MAKE30–90 (OR 0.76 [0.42–1.36]). For death, the stepwise approach demonstrated that SGLT2i use was associated with a reduced risk (OR 0.08; 0.01–0.64), and no effect was found for kidney replacement therapy (KRT). The subgroups of patients who experienced a reduction in the risk of MAKEs in patients with AKI treated with SGLT2is were those older than 61 years, those with an eGFR >81, and those without a history of hypertension or DM (*p* ≤ 0.05 for all).

**Conclusion:**

The use of SGLT2is during AKI had no effect on short- or medium-term MAKEs, but some subgroups of patients may have experienced benefits from SGLT2i treatment.

## Graphical Abstract

**Figure:**
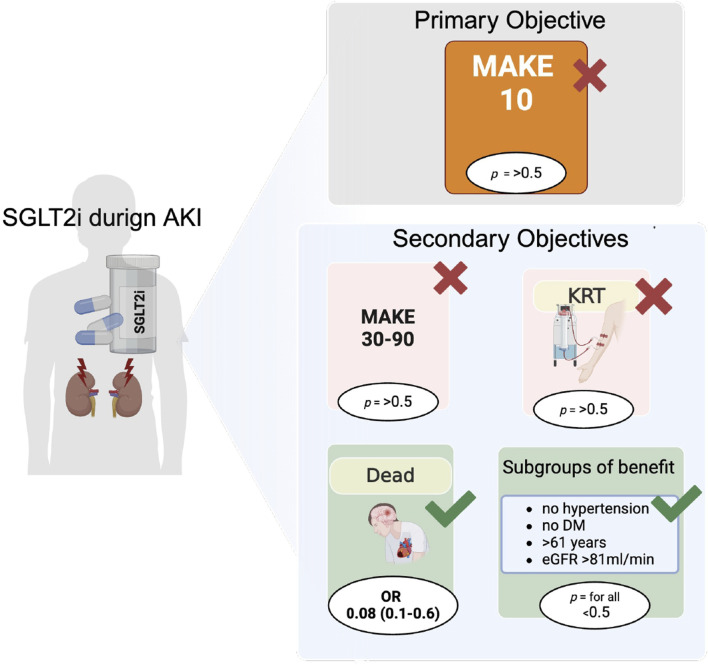

## Highlights

- Sodium–glucose cotransporter 2 inhibitors (SGLT2is) have revolutionized the treatment of chronic kidney disease (CKD).
- Their proven beneficial effects could improve renal function when administered during an AKI event.
- It would be very useful to know whether SGLT2is have some positive effects in patients with AKI.
- In this cohort of patients who were hospitalized with AKI, we observed that the use of SGLT2is during this period had no short- or medium-term effects on MAKEs but may be beneficial for some subgroups.
- The results may lead to a clinical trial in which patients with AKI are randomized to receive SGLT2is or placebo while monitoring medium-term MAKEs as the primary objective.

## Introduction

Sodium–glucose cotransporter 2 inhibitors (SGLT2is) have changed the treatment of chronic kidney disease (CKD) and have become one of the most relevant findings in the field of nephrology (Wright, 2021). Over the last decade, SGLT2is have been shown to reduce major kidney events (MAKEs) by 40% in people with or without diabetes (Kanda and Nangaku, 2019; Suzuki et al., 2022). Whether this benefit can be extended to other more specific kidney conditions, such as kidney transplantation (Pham and Pham, 2022), glomerular diseases such as IgA nephropathy, focal and segmental glomerulopathy (Morales and Galindo, 2022), acute cardiorenal syndrome (Schulze et al., 2022; Voors et al., 2022), or unusual pathologies such as Alport syndrome (Ge et al., 2023), and even to people on dialysis (Alhwiesh et al., 2022; De La Flor et al., 2023), is currently being explored. In specific scenarios, such as hospitalized critically ill patients or those with acute kidney injury (AKI), the nephrology community has been more cautious because SGLT2is are considered “sick day” drugs (Watson et al., 2023), which means that during critical illness or AKI, SGLT2is should not be administered or need to be suspended due to the reasonable risk of non-hyperglycemic ketoacidosis (Palmer and Clegg, 2021). Whether to suspend, continue, or start SGLT2is during AKI treatment has become an increasingly common question in daily clinical practice since patients who are usually treated with SGLT2is have a greater risk of developing AKI due to comorbidities such as diabetes or heart failure (Sawhney et al., 2020; Xu et al., 2020). AKI occurs frequently during hospitalization and occurs in up to 23% of critically ill patients, approximately 10% of whom require kidney replacement therapy (KRT), and approximately 50% of patients die during follow-up (Chávez-Íñiguez and Madero, 2022). Until now, there have been no specific treatments available for recovering kidney function after an episode of AKI or for reducing the mortality risk (Kashani et al., 2019). Prescribing SGLT2is during hospitalization in patients with AKI may improve kidney function through its mechanism of action, which, in theory, could protect the nephron, attenuate insults (Dekkers et al., 2018), and promote recovery (Castoldi et al., 2020; Bailey et al., 2022). Hence, there may be an association between the administration of SGLT2is during AKI and the incidence of MAKEs in the short and intermediate term. To fill this information gap, we conducted a retrospective cohort study comprising patients hospitalized with AKI who received SGLT2i treatment during their hospitalization to observe whether this treatment was associated with MAKEs.

## Methods

### Study design

This was a retrospective cohort study conducted at the Hospital Civil de Guadalajara Fray Antonio Alcalde, a tertiary referral academic center located in Mexico. All the patients included had AKI, received at least three consecutive doses of SGLT2is during hospitalization, and had sufficient data to analyze the MAKEs. In Mexico, there are only three SGLT2is available: dapagliflozin, empagliflozin, and canagliflozin, and patients treated with any of them were classified into the SGLT2i group. The SGLT2i administration data were collected by a physician who specifically looked for the prescription. AKI was diagnosed using the serum creatinine (sCr) KDIGO criteria, and CKD was defined as an estimated glomerular filtration rate (eGFR) of less than 60 mL/min/1.73 m^2^ for more than 3 months (Kellum et al., 2012). For AKI events, we chose only those patients who consulted the nephrology department. We selected the MAKE outcomes because of the recommendation to assess homogeneous results in studies conducted on AKI patients (Billings and Shaw, 2014). MAKEs were defined as death, a new requirement for dialysis, or worsening of kidney function by a ≥25% decline in the eGFR from baseline. We chose the MAKE10 criteria (i.e., the incidence of MAKEs within 10 days of follow-up) because most AKI patients start KRT and/or die during this timeframe (Kellum et al., 2012). Finally, for a total follow-up, we assessed MAKEs over 30–90 days (MAKE30–90) after the index event day (AKI hospitalization with or without SGLT2i). The patients included in the study had baseline sCr levels defined as the most recent sCr value in the last 6 months prior to hospitalization, and those who had sCr levels in the following months were included in the corresponding MAKE analyses. The exclusion criteria were AKI 3 months before hospitalization, CKD grade 5, chronic dialysis, hospital stay less than 48 h, kidney transplant, pregnancy, and missing data that would render the analysis incomplete.

The study was approved by the Hospital Civil de Guadalajara Fray Antonio Alcalde Institutional Review Board (HCG/CEI-0550/15) and was conducted in accordance with the Declaration of Helsinki. Informed consent was waived for the study. The study protocol adhered to the Strengthening the Reporting of Observational Studies in Epidemiology (STROBE) guidelines (von Elm et al., 2009) and the REporting of studies Conducted using Observational Routinely collected health Data (RECORD) statement (Benchimol et al., 2015).

### Data collection

Clinical characteristics, demographic information, and laboratory data were collected prospectively via automated retrieval from the institutional electronic medical record system. The baseline sCr level was defined as the most recent value within the 6 months prior to admission. Contributing factors of AKI include nephrotoxic drugs such as aminoglycosides, NSAIDs, vancomycin, and amphotericin B, as well as shock (administration of vasopressors for a mean arterial pressure <65 mmHg). We re-collected biochemical data such as the levels of hemoglobin, platelets, leukocytes, glucose, urea, sCr, sodium, potassium, chloride, phosphate, and calcium. The indications for KRT included fluid overload resistance to diuretics, severe hyperkalemia, severe metabolic acidosis, and uremic manifestations such as encephalopathy, pericarditis, and seizures (Kellum et al., 2012), (Negi et al., 2016), (Leaf and Waikar, 2019).

### Study outcomes and objectives

The purpose of this study was to investigate the association between the use of SGLT2is during an episode of AKI and MAKEs during a medium-term follow-up.

The primary objective was the risk of MAKEs during the first 10 days of follow-up (MAKE10). The secondary objectives were each individual MAKE contributor, such as KRT or death, and MAKEs during the medium follow-up period of 30–90 days (MAKE30–90). In addition, a stratification analysis for outcomes across different subgroups was performed, with a separate analysis for the diagnosis of DM, chronic heart failure (CHF), SAH, those with an eGFR >81 mL/min/1.73 m^2^, and age. These subgroups were considered since we believe that these variables, considered healthy patients, could influence this relationship. A search was carried out in the electronic record for the diagnosis of ketosis or ketoacidosis, trying to identify if SGLT2i consumption was associated with these events.

### Statistical analysis

The distribution of the quantitative variables was visually examined by histograms, and the Kolmogorov‒Smirnov and Shapiro‒Wilk tests were used to confirm their non-normal distribution. Continuous variables are expressed as medians and interquartile ranges, while categorical variables are expressed as counts and proportions. Differences in categorical variables between the SGLT2i and non-SGLT2i groups were analyzed using the χ2 test or Fisher’s exact test, as appropriate. Continuous variables were compared with the Wilcoxon rank test.

Logistic regression analysis was used to determine the risk of MAKE10, MAKE30–90, and the initiation of KRT in three different models. Model 1 was adjusted for variables with *p* < 0.1 in the analysis of differences between groups in terms of baseline characteristics and clinically relevant characteristics. For Model 2, we used a forward stepwise analysis, including every variable from the demographic characteristics and adding to the model those variables with *p* < 0.1. In Model 3, we included those variables that were statistically significant in both of the previously mentioned models and locked SGLT2i use as a categorical independent variable. The process was replicated for the primary outcome, MAKE10, and for MAKE30–90. For death and initiation of KRT, only the stepwise approach was used.

We estimated the average treatment effect by nearest-neighbor matching. Nearest-neighbor matching estimators impute the missing potential outcome for each subject using an average of the outcomes of similar subjects who receive the other exposure level. The similarity between the subjects was based on a weighted function of the covariates for each observation that included variables of Model 3 in logistic regression. The effects of SGLT2i use were estimated for MAKE10, MAKE30–90, and death. A stratification analysis with the calculation of odds ratios (ORs) for outcomes across different subgroups was performed. *p* < 0.05 indicates statistical significance. The data were analyzed using Stata version 16.1 (StataCorp, College Station, TX, United States of America).

## Results

**FIGURE 1:**
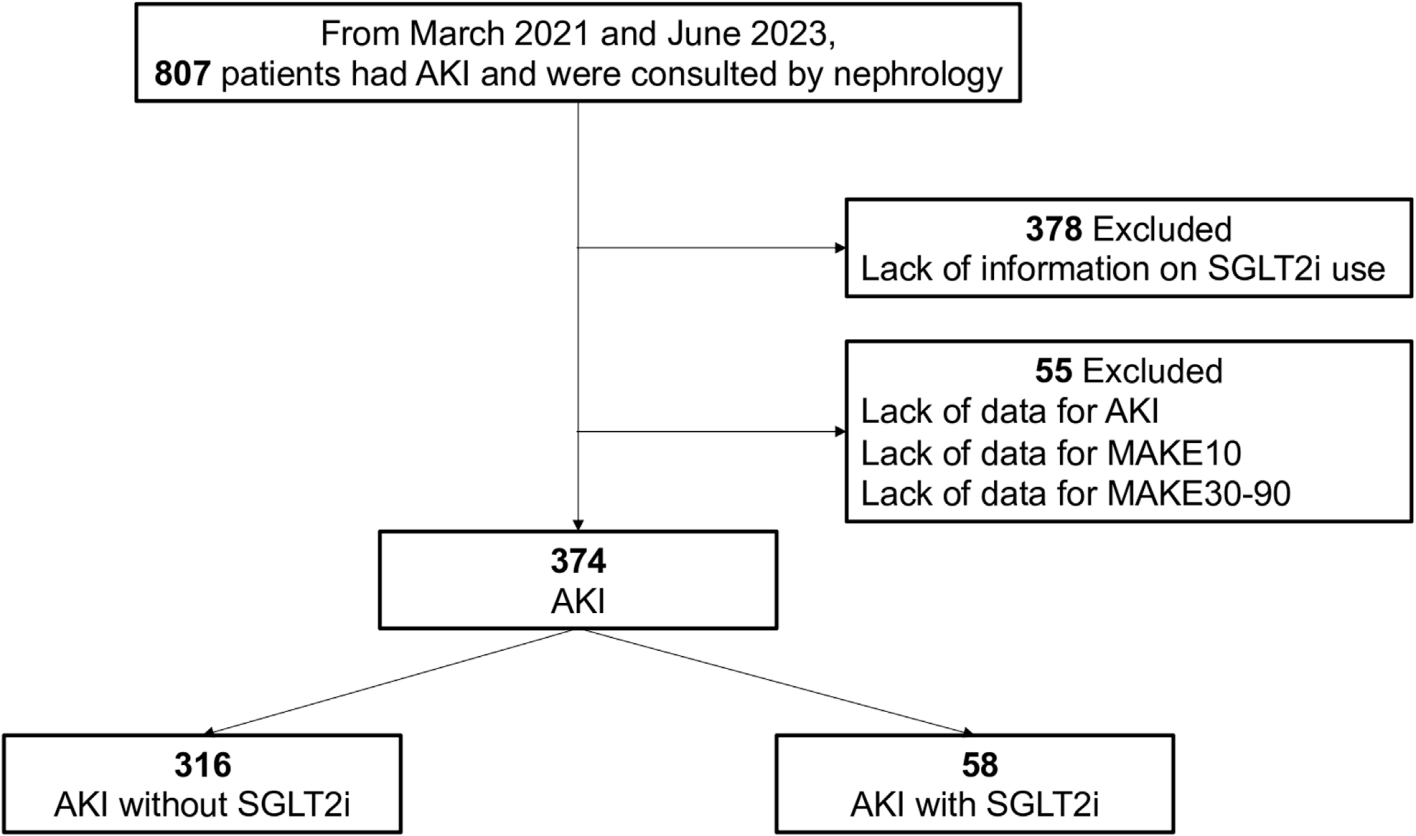
Flow chart of the study population selection process.

From March 2021 to June 2023, 807 patients had AKI and were referred to the nephrology department, and 378 patients were excluded for a lack of information. Hence, 429 patients were assessed as candidates for the study; 55 patients were excluded because of a lack of data on any of the outcomes. Ultimately, 374 patients were included in the analysis—58 and 316 patients with and without SGLT2i therapy, respectively. A flow chart of the study population is shown in Figure 1.

Table 1 describes the demographic and clinical characteristics of patients with AKI according to the use of SGLT2is. Significant differences were found among these groups. Considering the most relevant, we found that the patients who used SGLT2is, compared to those who did not, were older (62 vs. 53 years); had a greater prevalence of diabetes (58.6% vs. 29.7%), hypertension (70.6% vs. 32.3%), hypothyroidism (8.6% vs. 2.5%), chronic heart failure (41.3% vs. 6.6%), CKD (55.1% vs. 19.6%), and ischemic heart disease (31% vs. 2.2%); and had a lower eGFR (46.2 vs. 90.8 mL/min/1.73 m^2^) (*p* for all <0.05). Those who were treated with SGLT2is used diuretics more frequently and used fewer vasopressors and antibiotics. Among the most common etiologies of AKI, those who used SGLT2is had less frequent diagnoses of sepsis, hypovolemia, obstructive nephropathy, and, more commonly, cardiorenal syndrome. The SGLT2i group needed hemodialysis less often during hospitalization. These patients also presented with AKI stage 2 or 3 less frequently and had a 33% lower mortality rate than the patients without SGLT2is. Mortality was significantly lower in those who consumed SGLT2is (10% vs. 29%); this statistical difference was not observed in the frequency of presenting WRF (87% vs. 79%) (Table 1).

**TABLE 1:** Baseline clinical characteristics of AKI patients according to SGLT2i use.

Variable	AKI without SGLT2i	AKI with SGLT2i	Total	*p*-value
Demographic characteristics				
N	316	58	374	
Age [years], median (IQR)	53 (40–67)	62 (51–68)	55 (41–67)	<0.01
Male sex [N (%)]	192 (60.7)	32 (55.1)	224 (59.8)	0.42
Body mass index	23 (20.7–25.3)	23.5 (21–27.4)	23 (20.76–25.3)	0.40
Systolic BP [N]	114 (100–130)	115 (102–135)	114 (100–130)	0.55
Diastolic BP [N]	69 (60–78)	70 (62–80)	69 (60–79)	0.14
Diabetes [N (%)]	94 (29.7)	34 (58.6)	128 (34.2)	<0.01
Hypertension [N (%)]	102 (32.3)	41 (70.6)	143 (38.2)	<0.01
Hypothyroidism [N (%)]	8 (2.5)	5 (8.6)	13 (3.48)	0.02
Chronic heart failure [N (%)]	21 (6.6)	24 (41.3)	45 (12)	<0.01
Ischemic heart disease [N (%)]	7 (2.2)	18 (31)	25 (6.6)	<0.01
Baseline eGFR, mL/min/1.73m^2^	90.8 (47.9–97)	46.2 (30.7–67.2)	81.7 (42.7–96.8)	<0.01
Chronic kidney disease [N (%)]	62 (19.6)	32 (55.1)	94 (25.1)	<0.01
Sepsis [N (%)]	146 (46.2)	10 (17.2)	156 (41.7)	<0.01
Hypovolemia [N (%)]	87 (27.5)	8 (13.7)	95 (25.4)	0.02
Cardiorenal syndrome [N (%)]	24 (7.5)	34 (58.6)	58 (15.5)	<0.01
Nephrotoxic drugs [N (%)]	9 (2.8)	1 (1.7)	10 (2.6)	0.62
Shock [N (%)]	39 (12.3)	5 (8.6)	44 (11.7)	0.41
Obstructive nephropathy [N (%)]	48 (15.1)	2 (3.4)	50 (13.3)	0.01
Hemoglobin, gr/L, mean	9.81 (8.08–11.82)	10.52 (8–13.04)	9.86 (8.01–12.11)	0.30
Platelets, mean	171 (100–270)	196 (136–276)	179 (103–271)	0.17
Leucocytes, mean	13.1 (9.2–18.6)	10.15 (7.64–14.5)	12.6 (8.83–18.42)	0.01
Glucose, mg/dL, mean	114 (86–150)	106.5 (81–146)	114 (85–149)	0.20
Urea, mg/dL, mean	162 (112–223)	159.5 (119–210)	161.5 (115.6–221)	0.88
Creatinine, mg/dL	3.8 (2.4–5.7)	3.5 (2.5–5.8)	3.8 (2.4–5.78)	0.73
Sodium, mmol/L, mean	136 (131.140)	134 (131–137)	136 (131–140)	0.06
Potassium mmol/L, mean	4.7 (4.07–5.5)	4.63 (4.12–5.57)	4.7 (4.1–5.5)	0.75
NSAIDs [N (%)]	113 (35.7)	16 (27.5)	129 (34.4)	0.22
Antibiotics [N (%)]	253 (80)	27 (46.5)	280 (74.8)	<0.01
Antihypertensives [N (%)]	79 (25)	43 (74)	122 (32.6)	<0.01
Diuretics [N (%)]	113 (35.7)	39 (67.2)	152 (40.6)	<0.01
Vasopressor use [N (%)]	108 (34.18)	8 (13.7)	116 (31)	<0.01
Statins [N (%)]	30 (9.4)	37 (63.7)	67 (17.9)	<0.01
Acetylsalicylic acid [N (%)]	18 (5.7)	25 (43.1)	43 (11.5)	<0.01
Kidney replacement therapy				
Hemodialysis [N (%)]	87 (27.5)	8 (13.9)	95 (25.4)	0.02
Hyperkalemia [N (%)]	50 (15.8)	4 (6.9)	54 (14.4)	0.07
Metabolic acidosis [N (%)]	48 (15.1)	4 (6.9)	52 (13.9)	0.09
Fluid overload [N (%)]	31 (9.8)	8 (13.7)	39 (10.4)	0.36
Uremic syndrome [N (%)]	50 (15.8)	8 (13.7)	58 (15.5)	0.69
Medical treatment				
Fluid adjustment [N (%)]	197 (78.8)	42 (80.7)	239 (79.1)	0.75
Nephrotoxic withdrawal	22 (8.8)	1 (1.9)	23 (7.6)	0.08
Outcomes				
KDIGO-1 [N (%)]	15 (4.7)	5 (8.6)	20 (5.3)	<0.01
KDIGO-2 [N (%)]	35 (11)	3 (5.1)	38 (10.1)	
KDIGO-3 [N (%)]	222 (70.2)	19 (32.7)	241 (64.4)	
Acute-on-chronic kidney disease	44 (13.9)	31 (53.4)	75 (20)	
MAKEs at 10 days	251 (79.4)	43 (74.1)	294 (78.6)	0.36
MAKEs at 30–90 days	194 (61.3)	30 (51.7)	224 (59.8)	0.16
KRT at 10 days	120 (37.9)	15 (25.8)	135 (36.1)	0.07
Mortality at 10 days [N (%)]	94 (29.7)	6 (10.3)	100 (26.7)	<0.01
WRF	277 (87.6)	46 (79.3)	323 (86.3)	0.08

Data are presented in median (IQR) or proportion (%).

Abbreviations: CRRT, continuous renal replacement therapy; MAKE, major adverse kidney event; KRT, kidney replacement therapy; and WRF, worsening of renal function.

### Primary outcome: the association between SGLT2i use during AKI and the risk of MAKE10

**FIGURE 2:**
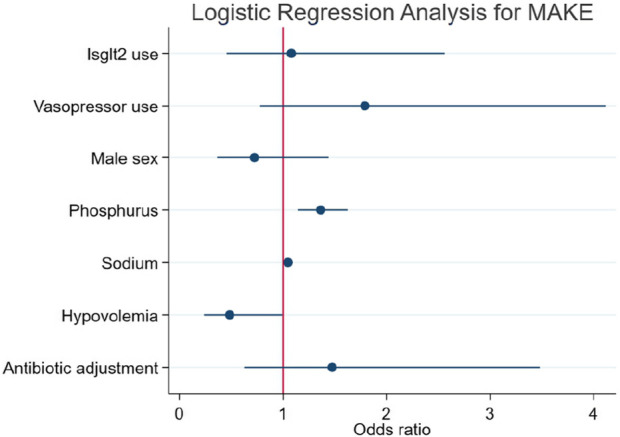
Logistic regression analysis for MAKE10 in patients according to the use or absence of SGLT2is.

MAKE10 was present in 78.6% of the total population and in 79.4% and 74.1% of patients without and with SGLT2is, respectively. With the aim of exploring the relationship between patients who have AKI and are treated with SGLT2is during hospitalization with MAKE10, a multiple-variable logistic regression was performed. In Model 1, a manual approach revealed that only vasopressors were associated with MAKE10, with an OR of 2.28 (1.00–5.19). In Model 2, a stepwise approach showed that male sex (OR 0.21; 0.08–0.55) and hypovolemia (OR 0.37; 0.14–0.96) attenuated the risk. In contrast, a phosphate OR of 1.44 (1.13–1.83) and a serum sodium OR of 1.07 (1.01–1.14) were associated with a greater risk of MAKE10. Finally, in Model 3, logistic regression analysis revealed that SGLT2i use was not associated with the risk of MAKE10 (OR 1.08 [0.45–2.56]), as reported in Table 2 and Figure 2.

**TABLE 2:** Logistic regression analysis for the primary outcome.

MAKE10	OR	LCI	UCI	P
Model 1 Manual approach				
SGLT2i use	0.579	0.210	1.599	0.292
Age	1.008	0.986	1.029	0.458
Body mass index	0.890	0.925	1.040	0.923
Diabetes mellitus	0.764	0.338	1.730	0.520
Systemic hypertension	1.179	0.493	2.819	0.711
Hypothyroidism	2.535	0.265	24.252	0.419
Congestive heart failure	0.734	0.254	2.124	0.569
Chronic kidney disease	0.898	0.377	2.135	0.808
Ischemic heart disease	0.616	0.141	2.692	0.520
Glomerular filtration rate	1.008	0.996	1.020	0.163
Use of antihypertensive	1.822	0.787	4.213	0.161
Use of diuretic	1.801	0.866	3.741	0.115
Vasopressor use	2.288	1.008	5.193	0.048
Sepsis	0.951	0.469	1.930	0.891
Hypovolemia	0.769	0.371	1.592	0.480
Model 2 Stepwise approach				
Male sex	0.211	0.080	0.559	0.002
Phosphate	1.440	1.131	1.833	0.003
Sodium	1.078	1.014	1.145	0.015
Hypovolemia	0.371	0.142	0.969	0.043
Antibiotic adjustment	3.078	0.952	9,946	0.060
Model 3 Final logistic regression model				
SGLT2i use	1.081	0.456	2.563	0.859
Vasopressor use	1.791	0.779	4.115	0.170
Male sex	0.724	0.364	1.441	0.359
Phosphate	1.365	1.148	1.624	<0.001
Sodium	1.049	1.005	1.094	0.026
Hypovolemia	0.486	0.237	0.994	0.048
Antibiotic adjustment	1.475	0.625	3.480	0.375

Abbreviations: CRRT, continuous renal replacement therapy; MAKE, major adverse kidney event; KRT, kidney replacement therapy; and WRF, worsening of renal function.

### Secondary outcomes: the association between SGLT2i use during AKI and the risk of MAKE30–90, death, and KRT

MAKE30–90 was present in 59.8% of the total population and in 61.3% and 51.7% of patients treated without and with SGLT2is, respectively.

**FIGURE 3:**
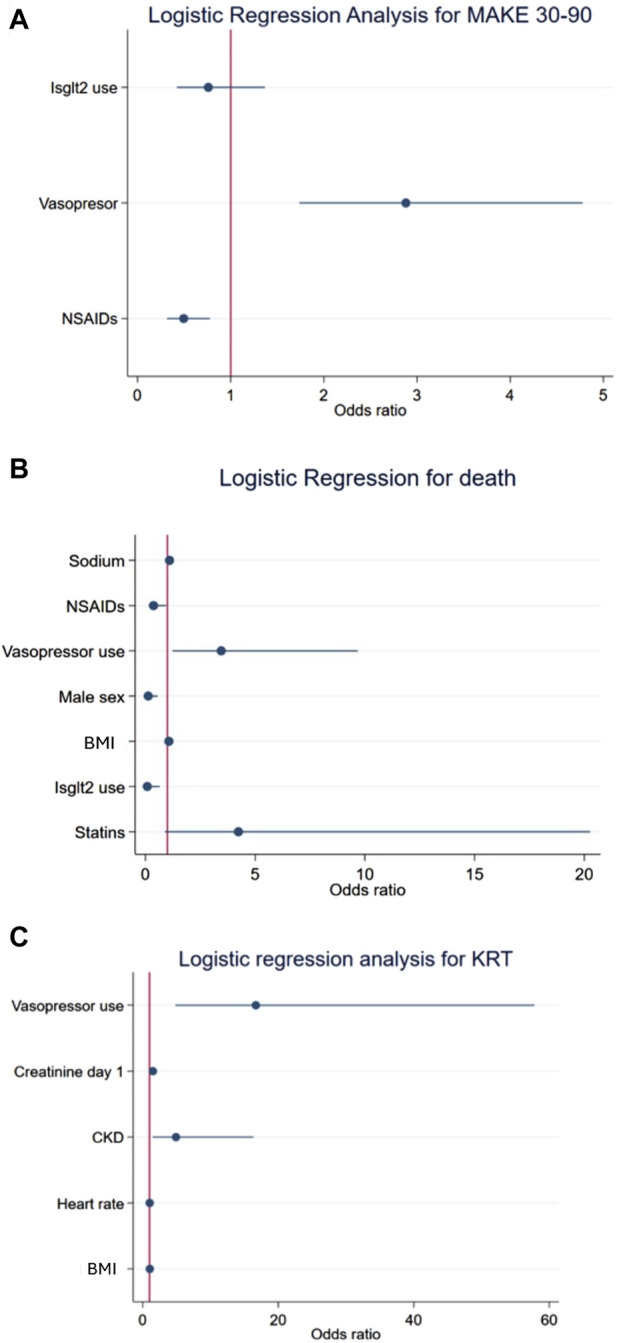
Logistic regression of the secondary endpoints: MAKE30–90 **(A)**, death **(B)**, and KRT **(C)**.

Table 3 and Figure 3 show the secondary objectives of the cohort. According to the manual approach, SGLT2i treatment was associated with a reduction in the risk of MAKE30–90 (OR 0.36; 0.135–0.972), an effect that was lost in the final logistic regression model (Figure 3A).

**TABLE 3:** Logistic regression analysis for secondary outcomes.

MAKE30–90	OR	LCI	UCI	P
Manual approach				
SGLT2i use	0.363	0.135	0.972	0.044
Age	1.001	0.982	1.021	0.855
Body mass index	0.937	0.963	1.032	0.913
Diabetes mellitus	1.088	0.519	2.281	0.822
Systemic hypertension	0.543	0.246	1.200	0.132
Hypothyroidism	1.784	0.340	9.355	0.493
Congestive heart failure	1.024	0.366	2.868	0.963
Chronic kidney disease	1.313	0.577	2.986	0.516
Ischemic heart disease	0.503	0.114	2.213	0.364
Glomerular filtration rate	0.997	0.986	1.008	0.670
Use of antihypertensive	1.884	0.869	4.086	0.108
Use of diuretic	1.112	0.587	2.105	0.743
Vasopressor use	2.988	1.446	6.173	0.003
Sepsis	0.755	0.395	1.442	0.396
Hypovolemia	0.833	0.424	1.638	0.598
Stepwise approach				
NSAIDs	0.262	0.116	0.590	0.001
Vasopressor use	5.850	1.970	17.370	0.001
Final logistic regression model				
SGLT2i use	0.760	0.422	1.366	0.359
Vasopressor use	2.879	1.736	4.775	<0.001
NSAIDs	0.495	0.316	0.776	0.002

Death	OR	LCI	UCI	P
Stepwise approach				
Sodium	1.096	1.028	1.168	0.005
NSAIDs	0.366	0.142	0.939	0.037
Vasopressor use	3.452	1.231	9.676	0.018
Male sex	0.124	0.028	0.545	0.006
SGLT2i use	0.081	0.010	0.642	0.017
Statin use	4.232	0.885	20.267	0.071

KRT	OR	LCI	UCI	P
Stepwise approach				
Vasopressor use	16.697	4.824	57.788	<0.001
Creatinine	1.506	1.202	1.886	<0.001
Chronic kidney disease	4.914	1.476	16.355	0.009
Heart rate	1.031	1.004	1.058	0.021

For death, the stepwise approach demonstrated that the variables associated with a reduced risk were the use of NSAIDs, male sex, and the use of SGLT2is. Sodium and the use of vasopressors were associated with an increased risk of death (Table 3; Figure 3B). The stepwise approach was used to assess the risk of KRT, and the variables associated with increased risk were the use of vasopressors, creatinine, CKD, and heart rate. The data are reported in Table 3 and Figure 3C.

### Nearest-neighbor matching

Due to the great heterogeneity of the population of patients with AKI who were treated with SGLT2is compared to those who were not, an analysis of nearest-neighbor matching was performed to assess the effect of SGLT2i use on MAKE10, MAKE30–90, and death. We also used covariates for the included variables of Model 3 in logistic regression, with a nearest-neighbor ratio of 2:1. A significant OR was observed for MAKE10 (OR 1.09, 1.00–1.19), and no effect was found for MAKE30–90, death, or KRT (*p* = ≤ 0.05 for all). A detailed description of the analysis is presented in Table 4.

**TABLE 4:** Effect of SGLT2i use on outcomes determined by nearest-neighbor matching.

Outcome	OR	LIQR	UIQR	*p*
MAKE10	1.094498754	1.003324815	1.193957835	0.042
MAKE30–90	0.890561604	0.672957568	1.178529058	0.417
Death	0.870389906	0.662672353	1.143217437	0.318
KRT	1.069707664	0.805469453	1.42063066	0.642

Adjustment for covariates primary diagnosis, age, BMI, diabetes mellitus, hypertension, hypothyroidism, congestive heart failure, chronic kidney disease, ischemic coronary disease, and vasopressor use.

### Subgroup analysis for the risk of MAKE10 and MAKE30–90 in patients with AKI by SGLT2i treatment status

**FIGURE 4:**
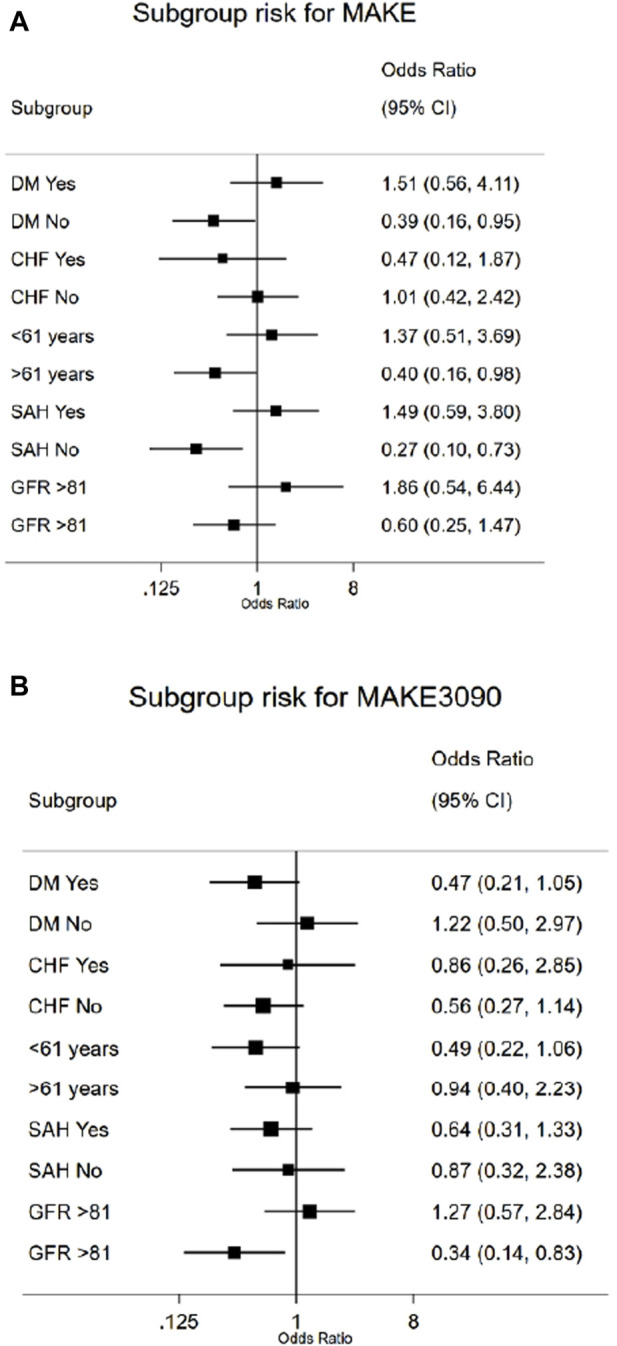
Forest plot analysis of the risk of MAKE10 **(A)** and MAKE30–90 **(B)** in patients in the SGLT2i subgroup.

The subgroups associated with the risk of MAKE10 were those without a history of diabetes, those >61 years old, and those who did not have a diagnosis of hypertension (*p* ≤ 0.05 for all) (Figure 4A). For the risk of MAKE30–90, we observed that patients with an eGFR >81 benefited the most from receiving SGLT2is, with a risk reduction of 66% (Figure 4B). No diagnoses of ketosis or ketoacidosis were identified in the electronic database.

## Discussion

In this retrospective cohort, we observed that in hospitalized patients with AKI, SGLT2i treatment was not associated with the risk of MAKE10 or MAKE30–90; however, it was revealed that some specific subgroups may benefit from SGLT2i use (Graphical Abstract).

We did not identify an immediate beneficial effect on the kidney for those patients who were hospitalized with AKI and treated with SGLT2is, as evaluated by MAKE10. These findings of no immediate beneficial effect on kidney function are in line with those presented in three cohorts, in which the effect of SGLT2is was explored during the hospitalization of critically ill patients with vulnerable kidneys or AKI. The first is the DARE-19 clinical trial, in which 1,250 hospitalized patients with moderate COVID-19 were randomized to receive dapagliflozin or placebo to determine the effects on a key secondary kidney outcome (composite of AKI, KRT, or death), and they found no benefit of SGLT2is (Heerspink et al., 2022). The second is a case-control study of 90 patients with diabetes hospitalized in the intensive care unit with an insulin pump. The patients who received empagliflozin did not experience a change in kidney function during their stay (Mårtensson et al., 2023). In the third case, in a cohort of patients with AKI, 356 patients with cardiorenal syndrome type 1 (74% had AKI stage 1) received SGLT2i treatment during hospitalization. There was no improvement in kidney function recovery, but SGLT2i was associated with a 55% reduction in the risk of death at the 30-day follow-up (Aklilu et al., 2023). Our findings add to the aforementioned studies, where SGLT2i treatment may be considered safe for patients at a high risk of kidney injury or in those with AKI; SGLT2is do not cause further deterioration of kidney function, and a positive effect may be found. Notably, compared to those studies, our cohort included patients with more comorbidities and variables associated with greater AKI severity, and we included only patients with AKI; 31% had shock, 64% had AKI stage 3, 25% started hemodialysis, and only 34% had diabetes, which explains our high mortality rate of approximately 26%.

It is plausible and reasonable to speculate that SGLT2is may have a positive effect on kidney function during an episode of AKI. There are multiple possible explanations for this effect, and some of the most studied mechanisms are the attenuation of the tubular hypoxic environment (Sano and Goto, 2019), the generation of an adaptive response to oxygen deprivation, the improved use of nutrients in tubular cells (Sasako et al., 2021), and a decrease in inflammatory factors such as KIM1 and IL-6 (Dekkers et al., 2018; Liu et al., 2021). SGLT2is have demonstrated efficiency in different scenarios and kidney pathologies that were unexpected, such as IgA nephropathy and focal and segmental glomerulosclerosis (Morales and Galindo, 2022). During AKI, these compounds exhibit renoprotective effects via enhanced ketogenesis, particularly through the amelioration of pathologically hyperactive mTORC1 signaling in damaged proximal tubular epithelial cells (Tomita et al., 2020), improved oxidative stress, fibrosis, and tubular atrophy (Packer, 2021). Through metabolomics, some benefits have been observed, such as improvements in endothelial function, energy metabolism, and mitochondrial function, and all of these mechanisms could also have a profound positive impact on kidney function (Mulder et al., 2020).

The expected decrease in the eGFR of approximately 5 mL/min/1.73 m^2^ in the first 4 weeks after starting SGLT2is (Meraz-Munoz et al., 2021) could be a reason for not starting SGLT2is in patients with AKI, considering that this could limit kidney recovery; however, in our study, we showed that this change does not occur. During AKI, SGLT2is do not have any impact on the recovery or deterioration of kidney function. Similarly to what was reported in two other studies, SGLT2is seem to be safe, as they do not cause new AKI episodes (Agarwal et al., 2022; Alkas et al., 2023). These relatively neutral effects contrast with the benefits reported by Pan et al. (2024), where patients with diabetes who survived hospitalization with AKI and who were treated with SGLT2is in the first 90 days after being discharged had a reduction in cardiorenal events and death.

Clinical trials of SGLT2is have consistently demonstrated an approximately 25% reduction in the risk of developing AKI in people with and without diabetes (Herrington et al., 2023), even in four meta-analyses of clinical trials (Gilbert and Thorpe, 2019; Menne et al., 2019; Zhao et al., 2020; Baigent, 2022). Additionally, in a cohort of more than 104,000 patients, previous use of SGLT2is also reduced the risk of starting KRT during AKI (Chung et al., 2023).

We found an association between reducing the risk of death in patients who were treated with SGLT2is. This result of the secondary objective is relevant and important. Our finding is in line with what was reported by Aklilu et al. (2023), who observed that patients with AKI who were previously treated with SGLT2is had an adjusted reduction of the risk of death (HR = 0.45, 95% CI = 0.23–0.87, *p* = 0.02), although the observed mortality risk reduction may be too large to attribute to SGLT2i exposure alone, and confounding by indication and baseline differences likely contributed to that finding. Evidence from experimental studies could explain why SGLT2i therapy can reduce the risk of death by rapidly improving endothelial function (Chung et al., 2023) and reducing myocardial oxidative stress-related injury and cardiac fibrosis (Irace et al., 2018). We believe that the relationship between mortality and the variables phosphate and vasopressors in AKI reflects a worse clinical status rather than a direct interaction with SGLT2is.

Because there were notable differences in the baseline characteristics of the patients with AKI who received SGLT2is, we performed nearest-neighbor matching to compare the groups more fairly. Subsequently, we found a slight but clinically irrelevant increase in the risk of MAKE10 in patients who used SGLT2is, but there were no increased risks detected for any of the other criteria considered.

We found certain subgroups of patients who may experience a protective effect against MAKEs with the use of SGLT2is during an episode of AKI, such as those without a history of hypertension or diabetes and those with a better eGFR (*p* ≤ 0.05). These findings are understandable since people considered to have better kidney function (i.e., those without CKD, diabetes, or hypertension) prior to an episode of AKI could have kidney tubules that are more amenable to SGLT2i benefits (Irace et al., 2018) in addition to greater renal reserve (Venkatachalam et al., 2015); therefore, SGLT2is may provide more benefit for some patients with AKI than for others, as has been demonstrated in meta-analyses of clinical trials (Kluger et al., 2019; Neuen et al., 2019; McGuire et al., 2021).

It is important to emphasize that SGLT2is have not been validated for use during an AKI episode, and caution should be taken in these scenarios due to their potential complications and adverse events.

The limitations of our study lie in its nature; as a retrospective cohort, we can demonstrate only associations and not causal relationships. In addition, the sample size was relatively small. Patients with multiple AKI etiologies may have different mechanisms of AKI initiation and recovery, which could affect the pathways targeted by SGLT2is. The characteristics of our patients, who were mostly critically ill patients with severe AKI, could limit the benefit of SGLT2i treatment during AKI. We tried to minimize the effect of baseline imbalance by adjusting for multiple potential confounders in multivariable models and matching group analysis, although it is likely that there are unmeasured baseline confounders contributing to an overestimation of the mortality benefit. Because we did not obtain a clear indication of the reason for the SGLT2i prescription, we could not determine whether that reason impacted MAKEs; for example, if SGLT2i therapy had been indicated during the decongestion of congestive heart failure (Salah et al., 2022) or for the treatment of hyperglycemia, the effect of that treatment on MAKEs may have differed. We do not know if SGLT2is were prescribed after hospital discharge, which could have impacted our secondary objectives. Finally, adverse events resulting from SGLT2i use were not systematically recorded; we reviewed the electronic records of patients who received SGLT2is and did not find any record of diabetic ketoacidosis (DKA) or ketosis. We believe that it is possible that some of these diagnoses could have occurred during the administration of SGLT2is, but their identification could have been omitted due to the presence of metabolic acidosis that can occur during AKI.

Our cohort study has several strengths, the first being that it is the only one of its kind to have considered only patients with AKI and to have monitored MAKEs during medium-term follow-up, which are outcomes considered appropriate for the study of the trajectory of AKI. A clinical trial is currently being carried out in patients with AKI who are randomized to receive an SGLT2i or a placebo to evaluate the transition to CKD (NCT05713851); these findings will surely contribute to a better understanding of the effects of this class of drugs in these highly vulnerable patients.

In conclusion, the use of SGLT2is during AKI had no effect on MAKEs in the short or medium term but may be beneficial in some patient subgroups. Our findings give rise to the design of a clinical trial in which SGLT2is are administered during AKI to evaluate their impact on MAKEs.

## Data Availability

Publicly available datasets were analyzed in this study. The files and data are in the physical and electronic archive of the Civil Hospital of Guadalajara Fray Antonio Alcalde and can be requested with prior authorization. All data generated or analyzed during this study are included in this article. Further inquiries can be directed to the corresponding author.
